# Animal Chemistry; or Organic Chemistry in Its Application to Physiology and Pathology

**Published:** 1842-11

**Authors:** 


### REVIEWS.

---

Art. IV.—*Animal Chemistry; or Organic Chemistry in its application to Physiology and Pathology.* By Justus Liebig, M. D., Professor of Chemistry in the University of Giessen. Edited from the Author's MS., by William Gregory, M. D., Professor of Chemistry, King's College, Aberdeen. With additions, notes, and corrections, by John W. Webster, M. D., Erving Professor of Chemistry in Harvard University. 1842.

Some one has termed Animal Chemistry the border territory lying between the domains of chemistry and physiology, which has been alternately claimed and occupied by both. Like all other border territories, the limits of which are not well defined, it has formed the theatre for warm and protracted controversies, each party claiming the right of governing it by his own laws. To the chemist, the processes of life have offered nothing more than examples of chemical affinity. In the stomach he has recognised a sort of fermenting vat, and in the lungs a furnace, where fuel is consumed and heat disengaged for warming the body. Some enthusiasts in this profession have not been able to see in all the mysterious functions of living beings any thing higher than a complicated and well arranged chemical laboratory, where composition and resolution, the generation of acids and alkalies, and the development of brain, blood-vessel and nerve go on under the direction of laws which govern the reactions of inorganic matter. The vitalist, on the other hand, has claimed for every action of the living organism the exclusive agency of the vital principle—rejecting all chemical influences as extraneous and hostile—asserting that chemistry and vitality are antagonist principles, each struggling for the ascendency—the vital force prevailing while the being lives, and only yielding up the body to chemistry when it dies. As in most controversies, truth, we apprehend, will be found to lie between the extremes of these opinions. The theories of the old chemists concerning the functions of vitality, it must be admitted, were visionary and absurd enough; but equally irrational must appear any attempt, at the present day, to solve many of the problems of vitality without a reference to chemistry. For the last twenty-five years physiology has been deriving the most important aid from the researches of the laboratory. Chemistry has improved its principles of research and greatly multiplied its facts, but never before did it receive so rich a contribution from this quarter as in the labors of Professor Liebig, from which we have no doubt a new era will be dated in the advance of physiology. In the work which stands at the head of this article the points of intersection of chemistry with physiology are clearly indicated, and it is shown that these sciences maintain towards each other relations similar to those which exist between chemistry and physics. In no work on animal chemistry has an attempt been made to explain so many vital processes upon chemical principles, and we are perfectly safe in asserting that no work has lately been given to the public, abounding in so many novel and original views, or which has excited in all quarters so startling an interest. When it was in progress it is said to have been submitted by its author to Müller, Tiedmann and Wagner, the three most distinguished physiologists in Germany, and while it is not pretended that they are pledged to the opinions of Professor Liebig, it is stated on high authority that there is but one feeling among them as to the vast importance of chemistry to physiology. We cannot, therefore, help regarding this ancient war as pretty nearly at an end. The physiologist and chemist, as their sciences in many parts are mixed up together, must go hand in hand in the investigation of the phenomena of life, for it is only by their joint labors that we can hope to attain a new physiology and a more rational pathology. Organic chemistry offers to the physiologist a sounding line which, although it is not long enough to measure the depths of the sea he is exploring, is yet the only instrument which can guide him in many of his investigations. The work before us, then, by far the ablest on that subject that has yet appeared, it will readily be believed, is full of interest for the medical man, and with that conviction we have undertaken to give in this article an analysis of its remarkable doctrines.

We will premise, that Dr. Liebig admits the existence of a vital principle, a *vis vitæ*, as distinct from electricity or chemical affinity, which is the source, in animals and vegetables, under the action of external influences, of growth and of reproduction, the subjects to which the first part of his work is devoted.

"We know not," he says, "how a certain something, invisible and imponderable in itself (heat), gives to certain bodies the power of exerting an enormous pressure on surrounding objects; we know not even how this something is produced when we burn wood or coals.

"So it is with the vital force, and with the phenomena exhibited by living bodies. The cause of these phenomena is not chemical force; it is not electricity, nor magnetism; it is a force which has certain properties in common with all causes of motion, and of change in form and structure in all material substances. It is a peculiar force, because it exhibits manifestations which are found in no other known force.

"As in the closed galvanic circuit, in consequence of certain changes which an inorganic body, a metal, undergoes when placed in contact with an acid, a certain something becomes cognizable by our senses, which we call a current of electricity; so, in the animal body, in consequence of transformations and changes undergone by matter previously constituting a part of the organism, certain phenomena of motion and activity are perceived, and these we call life or vitality."

Every motion or manifestation of force, according to our author, is the result of a transformation of the structure or substance of organs; every conception and mental affection is followed by changes in the chemical nature of the secreted fluids, and every thought and every sensation is accompanied by a change in the composition of the substance of the brain. The first conditions of animal life are nutritious matters and oxygen introduced into the system; and all vital activity arises from the reciprocal action of the oxygen and the elements of the food. At every moment of his life man is taking oxygen into his system from the atmosphere, in a quantity to amount, according to Lavoisier, to 746 lbs., and according to Menzies, to 837 lbs. in one year; and yet, if he has been supplied with sufficient food, we find his weight at the beginning and end of the year, if not quite the same, differing at most by only a few pounds. The question naturally occurs, what has become of the enormous weight of oxygen thus introduced in the course of a year into the human system?

"This question may be answered satisfactorily; no part of this oxygen remains in the system; but it is given out again in the form of a compound of carbon and hydrogen.

"The carbon and hydrogen of certain parts of the body have entered into combination with the oxygen introduced through the lungs and through the skin, and have been given out in the forms of carbonic acid gas and the vapor of water.

"At every moment, with every expiration, certain quantities of its elements separate from the animal organism, after having entered into combination, within the body, with the oxygen of the atmosphere.

"If we assume, with Lavoisier and Séguin, in order to obtain a foundation for our calculation, that an adult may receive into his system daily 32½ oz. (46,037 cubic inches=15,661 grains, French weight) of oxygen, and that the weight of the whole mass of his blood, of which 80 per cent. is water, is 24 lbs.; it then appears, from the known composition of the blood, that, in order to convert the whole of its carbon and hydrogen into carbonic acid and water, 64,103 grains of oxygen are required. This quanntity will be taken into the system of an adult in four days two hours.

"Whether this oxygen enters into combination with the elements of the blood, or with other other parts of the body containing carbon and hydrogen, in either case the conclusion is inevitable, that the body of a man, who daily takes into his system 32½ oz. of oxygen, must receive daily in the shape of nourishment, as much carbon and hydrogen as would suffice to supply 24 lbs. of blood with these elements; it being presupposed that the weight of the body remains unchanged, and that it retains its normal condition as to health."

This supply is furnished in the food, which must always be in a direct ratio to the quantity of oxygen taken into the system. Two animals respiring unequal quantities of oxygen will not require the same amount of nourishment; nor will an animal in a state of rest consume as much food as during exercise or work, the number of respirations in the latter case being greater than in the former. A child, whose respiration is more active than that of the adult, requires food oftener and is less patient of hunger. A bird, deprived of food, will die on the third day, while a reptile with its sluggish respiration will go half a year without food. Health suffers, whether the supply of food in proportion to the oxygen respired, be excessive or deficient. In one case obesity would be the result; and in the other dyspepsia and emaciation.

But the quantity of oxygen inspired is affected not only by the activity of the respiratory function, but also by the temperature and density of the atmosphere. In cold weather, and in cold climates, more air, and consequently more oxygen, enters the chest of a man than is respired by him in summer and in southern latitudes; and it is obvious that in an equal number of respirations we consume more oxygen at the level of the sea than on a mountain. The character and quantity of our food ought to correspond to these differences. In winter and in northern latitudes, more food is necessary, and food richer in carbon, upon which the oxygen acts. Certain savages, exposed almost naked to the severity of an arctic winter, will consume ten pounds of flesh, and "a dozen of tallow candles into the bargain," daily—facts which warmly clad travellers speak of with astonishment.

"Even when we consume equal weights of food in cold and warm countries, infinite wisdom has so arranged, that the articles of food in different climates are most unequal in the proportion of carbon they contain. The fruits on which the natives of the south prefer to feed do not in the fresh state contain more than 12 per cent. of carbon, while the bacon and train oil used by the inhabitants of the arctic regions contain from 66 to 80 per cent. of carbon.

"It is no difficult matter in warm climates, to study moderation in eating, and men can bear hunger for a long time under the equator; but cold and hunger united very soon exhaust the body.

"The mutual action between the elements of the food and the oxygen conveyed by the circulation of the blood to every part of the body is THE SOURCE OF ANIMAL HEAT."

It is well known that all animals possess within themselves a source of heat independent of surrounding objects. Even those animals called cold-blooded, maintain a temperature higher than the medium in which they live.

"This high temperature of the animal body, or, as it may be called, disengagement of heat, is uniformly and under all circumstances the result of the combination of a combustible substance with oxygen.

"In whatever way carbon may combine with oxygen, the act of combination cannot take place without the disengagement of heat. It is a matter of indifference whether the combination take place rapidly or slowly, at a high or at a low temperature; the amount of heat liberated is a constant quantity.

"The carbon of the food, which is converted into carbonic acid within the body, must give out exactly as much heat as if it had been directly burnt in the air or in oxygen gas; the only difference is, that the amount of heat produced is diffused over unequal times. In oxygen, the combustion is more rapid, and the heat more intense; in air it is slower, the temperature is not so high, but it continues longer.

"It is obvious, that the amount of heat liberated must increase or diminish with the quantity of oxygen introduced in equal times by respiration. Those animals which respire frequently, and consequently consume much oxygen, possess a higher temperature than others, which, with a body of equal size to be heated, take into the system less oxygen. The temperature of a child (102°) is higher than that of an adult (99.5°.) That of birds (104° to 105.4°) is higher than that of quadrupeds (98.5° to 100.4°) or than that of fishes or amphibia, whose proper temperature is from 2.7° to 3.6° higher than that of the medium in which they live. All animals, strictly speaking, are warm-blooded; but in those only which possess lungs is the temperature of the body quite independent of the surrounding medium."

The temperature of man is the same in all latitudes; yet the loss of heat at Palermo, where the external temperature is nearly equal to that of the body, and in the polar regions, where the temperature of the air is from 70° to 90° lower, must be very unequal, and the heat given off under the latter circumstances, must be restored within the body with great rapidity. This restoration is effected by the mutual action of the elements of the food and the inspired oxygen, which can never unite without the evolution of heat.

"In the animal body the food is the fuel; with a proper supply of oxygen we obtain the heat given out during its oxidation or combustion. In winter, when we take exercise in a cold atmosphere, and when consequently the amount of inspired oxygen increases, the necessity for food containing carbon and hydrogen increases in the same ratio; and by gratifying the appetite thus excited, we obtain the most efficient protection against the most piercing cold. A starving man is soon frozen to death: and every one knows that the animals of prey in the arctic regions far exceed in voracity those of the torrid zone.

"In cold and temperate climates, the air, which incessantly strives to consume the body, urges man to laborious efforts in order to furnish the means of resistance to its action, while, in hot climates, the necessity of labor to provide food is far less urgent.

"Our clothing is merely an equivalent for a certain amount of food. The more warmly we are clothed the less urgent becomes the appetite for food, because the loss of heat by cooling, and consequently the amount of heat to be supplied by the food, is diminished."

According to this view of the case, the quantity of food required by the system bears an exact ratio to the number of respirations, to the temperature of the air, and to the amount of heat given off to the surrounding medium. On this point Professor Liebig makes the following very interesting remarks:

"The Englishman in Jamaica sees with regret the disappearance of his appetite, previously a source of frequently recurring enjoyment; and he succeeds by the use of cayenne pepper and the most powerful stimulants, in enabling himself to take as much food as he was accustomed to eat at home. But the whole of the carbon thus introduced into the system is not consumed; the temperature of the air is too high, and the oppressive heat does not allow him to increase the number of respirations by active exercise, and thus to proportion the waste to the amount of food taken; disease of some kind, therefore, ensues.

"On the other hand, England sends her sick, whose diseased digestive organs have in a greater or less degree lost the power of bringing the food into that state in which it is best adapted for oxidation, and therefore furnish less resistance to the oxidizing agency of the atmosphere than is required in their native climate, to southern regions, where the amount of inspired oxygen is diminished in so great a proportion; and the result, an improvement in the health, is obvious. The diseased organs of digestion have sufficient power to place the diminished amount of food in equilibrium with the inspired oxygen; in the colder climate, the organs of respiration themselves would have been consumed in furnishing the necessary resistance to the action of the atmospheric oxygen.

"In our climate, hepatic diseases, or those arising from excess of carbon, prevail in summer; in winter, pulmonic diseases, or those arising from excess of oxygen, are more frequent."

The practical lesson to be drawn from this doctrine is obvious and impressive. Dr. Liebg has afforded a satisfactory explanation of a fact which observation has fully established, and by which men ought to have been taught long ago to adapt their diet to the circumstances of the climate in which they live.

Carbon has been specially alluded to as the element which, by uniting with oxygen, supports the temperature of the animal body, but we are not to lose sight of the fact, that the hydrogen of the food plays a part not less important than the carbon. The whole process of respiration, Professor Liebig remarks, is most clearly developed by the state of an animal wholly deprived of food.

"The first effect of starvation is the disappearance of fat, and this fat cannot be traced either in the urine or in the scanty fæces. Its carbon and hydrogen have been given off through the skin and lungs in the form of oxidized products; it is obvious that they have served to support respiration.

"In the case of a starving man, 32½ oz. of oxygen enter the system daily, and are given out again in combination with a part of his body. Currie mentions the case of an individual who was unable to swallow, and whose body lost 100 lbs. in weight during a month; and according to Martell (Trans. Linn. Soc., vol. xi. p. 411,) a fat pig, overwhelmed in a slip of earth, lived 160 days without food, and was found to have diminished in weight, in that time, more than 120 lbs. The whole history of hybernating animals, and the well established facts of the periodical accumulation, in various animals, of fat, which, at other periods, entirely disappears, prove that the oxygen, in the respiratory process, consumes, without exception, all such substances as are capable of entering into combination with it. It combines with whatever is presented to it; and the deficiency of hydrogen is the only reason why carbonic acid is the chief product; for, at the temperature of the body, the affinity of hydrogen for oxygen far surpasses that of carbon for the same element."

"In the progress of starvation, however, it is not only the fat which disappears, but also, by degrees, all such of the solids as are capable of being dissolved. In the wasted bodies of those who have suffered starvation, the muscles are shrunk and unnaturally soft, and have lost their contractility; all those parts of the body which were capable of entering into the state of motion, have served to protect the remainder of the frame from the destructive influence of the atmosphere. Toward the end, the particles of the brain begin to undergo the process of oxidation, and delirium, mania, and death close the scene; that is to say, all resistance to the oxidizing power of the atmospheric oxygen ceases, and the chemical process of eremacausis, or decay, commences, in which every part of the body, the bones excepted, enters into combination with oxygen.

"The time which is required to cause death by starvation depends on the amount of fat in the body, on the degree of exercise, as in labor or exertion of any kind, on the temperature of the air, and finally, on the presence or absence of water. Through the skin and lungs there escapes a certain quantity of water, and as the presence of water is essential to the continuance of the vital motions, its dissipation hastens death. Cases have occurred, in which a full supply of water being accessible to the sufferer, death has not occurred, till after the lapse of twenty days. In one case, life was sustained in this way for the period of sixty days.

"In all chronic diseases death is produced by the same cause, namely, the chemical action of the atmosphere. When those substances are wanting, whose function in the organism is to support the process of respiration; when the diseased organs are incapable of performing their proper function of producing these substances; when they have lost the power of transforming the food into that shape in which it may, by entering into combination with the oxygen of the air, protect the system from its influence, then, the substance of the organs themselves, the fat of the body, the substance of the muscles, the nerves, and the brain, are unavoidably consumed.*

"The true cause of death in these cases is the respiratory process, that is, the action of the atmosphere."

Professor Liebig next proceeds to examine the theory which ascribes to the nervous system the generation of animal heat. He remarks:

"No one will seriously deny the share which the nervous apparatus has in the respiratory process; for no change of condition can occur in the body without the nerves; they are essential to all vital motions. Under their influence, the viscera produce those compounds, which, while they protect the organism from the action of the oxygen of the atmosphere, give rise to animal heat, and when the nerves cease to perform their functions, the whole process of the action of oxygen must assume another form. When the pons Varolii is cut through in the dog, or when a stunning blow is inflicted on the back of the head, the animal continues to respire for some time, often more rapidly than in the normal state; the frequency of the pulse at first rather increases than diminishes, yet the animal cools as rapidly as if sudden death had occurred. Exactly similar observations have been made on the cutting of the spinal cord, and of the par vagum. The respiratory motions continue for a time, but the oxygen does not meet with those substances with which, in the normal state, it would have combined; because the paralyzed viscera will no longer furnish them. The singular idea that the nerves produce animal heat, has obviously arisen from the notion that the inspired oxygen combines with carbon, in the blood itself; in which case the temperature of the body, in the above experiments, certainly, ought not to have sunk. But, as we shall afterwards see, there cannot be a more erroneous conception than this.

"As by the division of the pneumogastric nerves the motion of the stomach and the secretion of the gastric juice are arrest-

*For an account of what really takes place in this process, I refer to the considerations on the means by which the change of matter is effected in the body of the carnivora, which will be found further on.

ed, and an immediate check is thus given to the process of digestion, so the paralysis of the organs of vital motion in the abdominal viscera affects the process of respiration. These processes are most intimately connected; and every disturbance of the nervous system or of the nerves of digestion reacts visibly on the process of respiration."

Our author proves by the most indisputable calculations, that the heat evolved during the union of oxygen with the elements of the food is quite sufficient to maintain the temperature of the animal body. Why, then, look further for other mysterious sources of caloric?

"According to the experiments of Despretz, 1 oz. of carbon evolves, during its combustion, as much heat as would raise the temperature of 78.15 oz. of water at 32° to 212°, that is, by 180 degrees; in all, therefore, 78.15 times 180°=14067 degrees of heat. Consequently, the 13.9 oz. of carbon which are daily converted into carbonic acid in the body of an adult, evolve 13.9+14067°=195531.3 degrees of heat. This amount of heat is sufficient to raise the temperature of 1 oz. of water by that number of degrees, or from 32° to 195563.3°; or to cause 67.9 lbs. of water at 32° to boil; or to heat 184.3 lbs. of water to 98.3° (the temperature of the human body;) or to convert into vapor 11.4 lbs. of water at 98.3°.

"If we now assume that the quantity of water vaporized through the skin and lungs in 24 hours amounts to 48 oz. (3 lbs.,) then there will remain, after deducting the necessary amount of heat, 144137.7 degrees of heat, which are dissipated by radiation, by heating the expired air, and in the excrementitious matters.

"In this calculation, no account has been taken of the heat evolved by the hydrogen of the food, during its conversion into water by oxidation within the body. But if we consider that the specific heat of the bones, of fat, and of the organs generally, is far less than that of water, and that consequently they require, in order to be heated to 98.3°, much less heat than an equal weight of water, no doubt can be entertained, that when all the concomitant circumstances are included in the calculation, the heat evolved in the process of combustion, to which the food is subjected in the body, is amply sufficient to explain the constant temperature of the body as well as the evaporation from the skin and lungs."

This explanation of the source of animal temperature is not peculiar to Dr. Liebig, but he has presented the doctrine in the clearest light, and supported it by an array of facts and arguments which appear to us to have settled the question.

On the subject of *reproduction* the author throws out the following hints, which are so curious that we are tempted to quote them:

"In a young animal the waste is less than the increase; and the female retains, up to a certain age, this peculiar condition of a more intense vegetative life. This condition does not cease in the female as in the male, with the complete development of all the organs of the body.

"The female in the lower animals, is, at certain seasons, capable of reproduction of the species. The vegetative life in her organism is rendered more intense by certain external conditions, such as temperature, food, &c.; the organism produces more than is wasted, and the result is the capacity of reproduction.

"In the human species, the female organism is independent of those external causes which increase the intensity of vegetative life. When the organism is fully developed, it is at all times capable of reproduction of the species; and infinite wisdom has given to the female body the power, up to a certain age, of producing all parts of its organization in greater quantity than is required to supply the daily waste.

"This excess of production can be shown to contain all the elements of a new organism; it is constantly accumulating, and is periodically expelled from the body until it is expended in reproduction. This periodical discharge ceases when the ovum has been impregnated, and from this time every drop of the superabundant blood goes to produce an organism like that of the mother.

"Exercise and labor cause a diminution in the quantity of the menstrual discharge; and when it is suppressed in consequence of disease, the vegetative life is manifested in a morbid production of fat. When the equilibrium between the vegetative and nervous life is disturbed in the male, when, as in eunuchs, the intensity of the latter is diminished, the predominance of the former is shown in the same form, in an increased deposit of fat."

The blood supplies the materials for the growth of the system and for repairing its waste. No substance, therefore, can be considered nutritious which is not susceptible of conversion into blood. The two most important ingredients of the blood, the fibrine and albumen, have very unexpectedly been proved to be identical in composition. They contain the elements out of which the living frrame is built up—nitrogen, phosphorus, sulphur, the earth of bones, sea salt and other salts of soda and potash, carbonic, phosphoric and sulphuric acids. The red globules with fibrine and albumen contain iron as a constant element. Fatty bodies, of a peculiar kind, are also found in the blood, and water forms its largest ingredient.

"Both the albumen and fibrine, in the process of nutrition, are capable of being converted into muscular fibre, and muscular fibre is capable of being reconverted into blood. These facts have long been established by physiologists, and chemistry has merely proved that these metamorphoses can be accomplished under the influence of a certain force, without the aid of a third substance, or of its elements, and without the addition of any foreign element, or the separation of any element previously present in these substances.

"If we now compare the composition of all organized parts with that of fibrine and albumen, the following relations present themselves—

"All parts of the animal body which have a decided shape, which form parts of organs, contain nitrogen. No part of an organ which possesses motion and life, is destitute of nitrogen; all of them contain likewise carbon and the elements of water, the latter, however, in no case in the proportion to form water.

"The chief ingredients of the blood contain nearly 17 per cent. of nitrogen, and no part of an organ contains less than 17 per cent. of nitrogen.

"The most convincing experiments and observations have proved that the animal body is absolutely incapable of producing an elementary body, such as carbon or nitrogen, out of substances which do not contain it; and it obviously follows, that all kinds of food fit for the production either of blood, or of cellular tissue, membranes, skin, hair, muscular fibre, &c., must contain a certain amount of nitrogen, because that element is essential to the composition of the above named organs; because the organs cannot create it from the other elements presented to them; and, finally, because no nitrogen is absorbed from the atmosphere in the vital process.

"The substance of the brain and nerves contains a large quantity of albumen, and, in addition to this, two peculiar fatty acids, distinguished from other fats by containing phosphorus (phosphoric acid?) One of these contains nitrogen (Frémy)."

In carnivorous animals the process of nutrition is seen in its simplest form. They live upon substances identical with their own flesh and blood, and in a chemical sense it may be said that, in supporting the vital process, they consume themselves. All their food is derived from blood, and being dissolved in the stomach, it is again, in its passage through the system, converted into blood, from which is reproduced all those parts of their organization which have undergone change or metamorphosis.

"The process of nutrition in graminivorous animals appears at first sight altogether different. The digestive organs are less simple, and their food consists of vegetables, the great mass of which contains but little nitrogen.

"From what substances, it may be asked, is the blood formed, by means of which their organs are developed? This question may be answered with certainty.

"Chemical researches have shown, that all such parts of vegetables as can afford nutriment to animals contain certain constituents which are rich in nitrogen; and the most ordinary experience proves that animals require for their support and nutrition less of these parts of plants in proportion as they abound in the nitrogenized constituents. Animals cannot be fed on matters destitute of these nitrogenized constituents.

These important products of vegetation are especially abundant in the seeds of the different kinds of grain, and of pease, beans and lentils; in the roots and the juices of what are commonly called vegetables. They exist, however, in all plants, without exception, and in every part of plants in larger or smaller quantity,"

These substances are *vegetable fibrine, vegetable albumen,* and *vegetable caseine,* nitrogonized compounds, out of which the blood of the herbivora is formed. Professor Liebig remarks:

"The chemical analysis of these three substances has led to the very interesting result that they contain the same organic elements, united in the same proportion by weight; and, what is still more remarkable, that they are identical in composition with the chief constituents of blood, animal fibrine, and albumen. They all three dissolve in concentrated muriatic acid with the same deep purple color, and even in their physical characters, animal fibrine and albumen are in no sense different from vegetable fibrine and albumen. It is especially to be noticed, that by the phrase identity of composition, we do not here imply mere similarity, but that even in regard to the presence and relative amount of sulphur, phosphorus, and phosphate of lime, no difference can be observed."

Vegetables, therefore, produce the blood of animals, for the carnivora, in consuming the graminivora, derive only such vegetable principles as had served for their nourishment. The vegetable fibrine and albumen which made the blood of the graminivorous animals, enter into the system of the carnivorous animal to constitute his flesh and blood. The development of the animal organism can only take place by the reception of certain principles identical with the chief ingredients of the blood. The blood it cannot create, but only receives it from substances which contain its chief constituents and gives to it its peculiar form. Out of the blood it produces nervous matter, cellular tissue, muscle, tendon and the various other parts which make up the animal frame, and which vegetables cannot produce.

But, besides these elements of food, rich in nitrogen, which contribute chiefly to the growth of animals, there are found other substances, as sugar, starch, and gum, necessary to the support of the animal system. The herbivorous animals quickly perish unless these compounds be supplied. What is the function performed by them? According to Professor Liebig, they cannot be employed in the production of blood, because the azotized substances of the food contain exactly the amount of carbon required for the formation of fibrine and albumen, and they must, therefore, be expended in the production of animal heat. The young of carnivorous animals find the requisite supply of nitrogenized food in the caseine of the the milk, which, curiously enough, turns out on analysis to be identical with the chief constituents of blood, fibrine and albumen—nay more, to be identical in all its properties with vegetable caseine. The caseine is converted into blood without the agency of any foreign substance, and the butter and the sugar of milk, containing no nitrogen, and only carbon, hydrogen and oxygen, become fuel for the maintenance of animal heat. The author proceeds:

"In the preceding pages it has been assumed, that the elements of the food are converted by the oxygen absorbed in the lungs into oxidized products; the carbon into carbonic acid, the hydrogen into water, and the nitrogen into a compound containing the same elements as carbonate of ammonia.

"This is only true in appearance; the body, no doubt, after a certain time, acquires its original weight. The amount of carbon, and of the other elements, is not found to be increased —exactly as much carbon, hydrogen, and nitrogen has been given out as was supplied in their food; but nothing is more certain than that the carbon, hydrogen, and nitrogen given out, although equal in amount to what is supplied in that form, do not directly proceed from the food.

"It would be utterly irrational to suppose that the necessity of taking food, or the satisfying the appetite, had no other object than the production of urea, uric acid, carbonic acid, and other excrementitious matters—of substances which the system expels, and consequently applies to no useful purposes in the economy.

"In the adult animal, the food serves to restore the waste of matter; certain parts of its organs have lost the state of vitality, have been expelled from the substance of the organs, and have been metamorphosed into new combinations, which are amorphous and unorganized.

"The food of the carnivora is at once converted into blood; out of the newly-formed blood those parts of organs which have undergone metamorphoses are reproduced. The carbon and nitrogen of the food thus become constituent parts of organs.

"Exactly as much carbon and nitrogen is supplied to the organs by the blood, that is, ultimately, by the food, as they have lost by the transformation attending the exercise of their functions.

"What then, it may be asked, becomes of the new compounds produced by the transformations of the organs, of the muscles, of the membranes and cellular tissue, of the nerves and brain?"

These compounds cannot be employed for the reproduction of those tissues from which they are derived, and if allowed to accumulate in the veins and lymphatics, they would speedily put a stop to the nutritive process. They are removed by an admirable contrivance thus described by Professor Liebig:

"The venous blood, before reaching the heart, is made to pass through the liver; the arterial blood, on the other hand, passes through the kidneys; and these organs separate from both all substances incapable of contributing to nutrition.

"Those new compounds wich contain the nitrogen of the transformed organs are collected in the urinary bladder, and being utterly incapable of any further application in the system, are expelled from the body.

"Those, again, which contain the carbon of the transformed tissues, are collected in the gall-bladder in the form of a compound of soda, *the bile*, which is miscible with water in every proportion, and which, passing into the duodenum, mixes with chyme. All those parts of the bile which, during the digestive process, do not lose their solubility, return during that process into the circulation in a state of extreme division. The soda of the bile, and those highly carbonized portions which are not precipitated by a weak acid (together making 99-100ths of the solid contents of the bile), retain the capacity of resorption by the small and large intestines; nay, this capacity has been directly proved by the administration of enemata containing bile, the whole of the bile disappearing with the injected fluid in the rectum.

"Thus we know with certainty, that the nitrogenized compounds, produced by the metamorphosis of organized tissues, after being separated from the arterial blood by means of the kidneys, are expelled from the body as utterly incapable of further alteration; while the compounds rich in carbon, derived from the same source, return into the system of carnivorous animals.

"The food of the carnivora is identical with the chief constituents of their bodies, and hence the metamorphoses which their organs undergo must be the same as those which, under the influence of the vital force, take place in the matters which constitute their food.

"The flesh and blood consumed as food yield their carbon for the support af the respiratory process, while its nitrogen appears as uric acid, ammonia, or urea. But previously to these final changes, the dead flesh and blood become living flesh and blood, and it is, strictly speaking, the carbon of the compounds formed in the metamorphoses of living tissues that serves for the production of animal heat.

"The food of the carnivora is converted into blood, which is destined for the reproduction of organized tissues; and by means of the circulation a current of oxygen is conveyed to every part of the body. The globules of the blood, which in themselves can be shown to take no share in the nutritive process, serve to transport this oxygen, which they give up in their passage through the capillary vessels. Here the current of oxygen meets with the compounds produced by the transformation of the tissues, and combines with their carbon to form carbonic acid, with their hydrogen to form water. Every portion of these substances which escapes this process of oxidation is sent back into the circulation in the form of the bile, which by degrees completely disappears.

"In the carnivora the bile contains the carbon of the metamorphosed tissues; this carbon disappears in the animal body, and the bile likewise disappears in the vital process. Its carbon and hydrogen are given out through the skin and lungs as carbonic acid and water; and hence it is obvious that the elements of the bile serve for respiration and for the production of animal heat. Every part of the food of carnivorous animals is capable of forming blood; their excrements, excluding the urine, contain only inorganic substances, such as phosphate of lime; and the small quantity of organic matter which is found mixed with these is derived from excretions, the use of which is to promote their passage through the intestines, such as mucus. These excrements contain no bile and no soda: for water extracts from them no trace of any substance resembling bile, yet bile is very soluble in water, and mixes with it in every proportion.

"Physiologists can entertain no doubt as to the origin of the constituent parts of the urine and of the bile. When, from deprivation of food, the stomach contracts itself so as to resemble a portion of intestine, the gall-bladder, for want of the motion which the full stomach gives to it, cannot pour out the bile it contains; hence in animals starved to death, we find the gall-bladder distended and full. The secretion of bile and of urine goes on during the winter sleep of hybernating animals; and we know that the urine of dogs, fed for three weeks, exclusively on pure sugar, contains as much of the most highly nitrogenized constituent, urea, as in the normal condition."

Professor Liebig rejects the idea that the bile is intended merely to be excreted. The following are his observations on this subject:

"Were the bile intended merely for excretion, we should find it more or less altered, and also the soda it contains, in the solid excrements. But, with the exception of common salt and of sulphate of soda, which occur in all the animal fluids, only mere traces of soda are to be found in the fæces. The soda of the bile, therefore, at all events, must have returned from the intestinal canal into the organism, and the same must be true of the organic matters which were in combination with it.

"According to the observations of physiologists, a man secretes daily from 17 to 24 oz. of bile; a large dog, 36 oz.; a horse 37 lbs. (Burdach's Physiologie, V. p. 260.) But the fæces of a man do not on an average weigh more than 5½ oz.; and those of a horse 28½ lbs., of which 21 lbs. are water, and 7½ lbs. dry fæces. (Boussingault.) The latter yield to alcohol only 1-20th part of their weight of soluble matter.

"If we assume the bile to contain 90 per cent. of water, a horse secretes daily 592 oz. of bile, containing 59.2 oz. of solid matter, while 7½ lbs. or 120 oz. of dried excrement yield only 6 oz. of matter soluble in alcohol, which might possibly be bile. But this matter is not bile; when the alcohol is dissipated by evaporation, there remains a soft, unctuous mass, altogether insoluble in water, and which, when incinerated, leaves no alkaline ashes, no soda.

During the digestive process, therefore, the soda of the bile, and, along with it, all the soluble parts of that fluid, are returned into the circulation. This soda reappears in the newly formed blood, and, finally, we find it in the urine in the form of phosphate, carbonate, and hippurate of soda. Berzelius found in 1000 parts of fresh human fæces only 9 parts of a substance similar to bile; 5 ounces, therefore, would contain only 21 grains of dried bile, equivalent to 210 grains of fresh bile. But a man secretes daily from 9,640 to 11,520 grains of fluid bile, that is, from 45 to 56 times as much as can be detected in the matters discharged by the intestinal canal."

And the results at which he arrives are thus stated:

"It cannot be disputed, that in an adult carnivorous animal, which neither gains nor loses weight perceptibly from day to day, its nourishment, the waste of organized tissue, and its consumption of oxygen, stand to each other in a well-defined and fixed relation.

"The carbon of the carbonic acid given off, with that of the urine; the nitrogen of the urine, and the hydrogen given off as ammonia and water; these elements, taken together, must be exactly equal in weight to the carbon, nitrogen, and hydrogen of the metamorphosed tissues; and since these last are exactly replaced by the food, to the carbon, nitrogen, and hydrogen of the food. Were this not the case, the weight of the animal could not possibly remain unchanged."

The fact has been alluded to that herbivorous animals consume matters containing no nitrogen, and destined for other purposes than the production of blood. In this class of animals the metamorphosis of existing tissues goes on much less rapidly than in the carnivora, and hence the necessity for materials of aliment which afford to their systems the elements for uniting with oxygen. Were the metamorphosis as rapid as in the systems of carnivorous animals, pastures a thousand times more luxuriant than the existing ones would scarcely suffice for their nourishment; and in that case sugar, gum, and starch would no longer be necessary to their sustenance, because the products of the waste of the organized tissues would contain enough carbon to support the respiratory function.

"Man, when confined to animal food, requires for his support and nourishment extensive sources of food, even more widely extended than the lion and tiger, because, when he has the opportunity, he kills without eating.

A nation of hunters, on a limited space, is utterly incapable of increasing in its numbers beyond a certain point, which is soon attained. The carbon necessary for respiration must be obtained from the animals, of which only a limited number can live on the space supposed. These animals collect from plants the constituents of their organs and of their blood, and yield them, in turn, to the savages who live by the chase alone. They, again, receive this food unaccompanied by those compounds, destitute of nitrogen, which, during the life of the animals, served to support the respiratory process. In such men, confined to an animal diet, it is the carbon of the flesh and of the blood which must take the place of starch and sugar.

"But 15 lbs. of flesh contain not more carbon than 4 lbs. of starch, and while the savage with one animal and an equal weight of starch could maintain life and health for a certain number of days, he would be compelled, if confined to flesh, in order to procure the carbon necessary for respiration, during the same time, to consume five such animals.

"It is easy to see, from these considerations, how close the connection is between agriculture and the multiplication of the human species. The cultivation of our crops has ultimately no other object than the production of a maximum of those substances which are adapted for assimilation and respiration, in the smallest possible space. Grain and other nutritious vegetables yield us, not only in starch, sugar, and gum, the carbon which protects our organs from the action of oxygen, and produces in the organism the heat which is essential to life, but is also in the form of vegetable fibrine, albumen, and caseine, our blood, from which the other parts of our body are developed.

"Man, when confined to animal food, respires, like the carnivora, at the expense of the matters produced by the metamorphosis of organized tissues; and, just as the lion, tiger, hyæna, in the cages of a menagerie, are compelled to accelerate the waste of the organized tissues by incessant motion, in order to furnish the matter necessary for respiration, so, the savage, for the very same object is forced to make the most laborious exertions and go through a vast amount of muscular exercise. He is compelled to consume force merely in order to supply matter for respiration."

The metamorphosis of tissues appears in the urine, which shows the waste in the carnivora to be much greater than in the graminivora. It is a novel thought of our author, that the phosphoric acid thus resulting goes to the production of brain in this latter class of animals. His words are,

"In the graminivora, therefore, whose food contains so small a proportion of phosphorus or of phosphates, the organism collects all the soluble phosphates produced by the metamorphosis of tissues, and employs them for the development of the bones and of the phosphorized constituents of the brain; the organs of excretion do not separate these salts from the blood. The phosphoric acid which, by the change of matter, is separated in the uncombined state, is not expelled from the body as phosphate of soda; but we find it in the solid excrements in the form of insoluble earthy phosphates."

In this class of animals the intensity of vegetative life far exceeds that of the carnivora. The cat will eat the first mouse presented to her, and perhaps the second, but even if she kills a third, she does not devour it. The cow and the horse eat almost without interruption from morning till night, and their systems convert into organized tissues all the food they consume, beyond the quantity required for merely supplying the waste of their bodies. They grow fleshy and plump, while the flesh of the carnivorous animal is always tough and sinewy. In wild animals of the herbivorous class, owing to the great exercise they take, oxygen enough is absorbed by respiration to consume the carbon of the gum, sugar and starch, and they remain comparatively lean. With our domestic animals the case is far different.

"The stall-fed animal eats, and reposes merely for digestion. It devours in the shape of nitrogenized compounds far more food than is required for reproduction, or the supply of waste alone; and at the same time it eats far more of substances devoid of nitrogen than is necessary merely to support respiration and to keep up animal heat. Want of exercise and diminished cooling are equivalent to a deficient supply of oxygen; for when these circumstances occur, the animal absorbs much less oxygen than is required to convert into carbonic acid the carbon of the substances destined for respiration. Only a small part of the excess of carbon thus occasioned is expelled from the body in the horse and ox, in the form of hippuric acid; and all the remainder is employed in the production of a substance, which, in the normal state, only occurs in small quantity as a constituent of the nerves and brain. This substance is *fat.*"

Now, it is a most curious fact, as stated by Prof. Liebig, that if we compare the composition of sugar and starch with that of mutton and beef suet and of human fat, we find that in all of them the proportion of carbon to hydrogen is the same, and that they only differ in that of oxygen, so that sugar, starch and gum, by the mere separation of a part of their oxygen, may pass into fat. The herbs consumed by the cow contain no butter, and no lard can be found in the corn and potatoes on which hogs are fatted, but these deposits are created in the organism of the animals, and, if the views of Prof. L. be correct, by the separation of oxygen from the elements with which it is combined in the food of the animals.

"Since the carbon of the fatty constituents of the animal body is derived from the food, seeing that there is no other source whence it can be derived, it is obvious if we suppose fat to be formed from albumen, fibrine, or caseine, that, for every 120 equivalents of carbon deposited as fat, 26 equivalents of oxygen must be separated from the elements of these substances; and further, if we conceive fat to be formed from starch, sugar, or sugar of milk, that for the same amount of carbon there must be separated 90, 100, and 110 equivalents of oxygen from these compounds respectively.

"There is, therefore, but one way in which the formation of fat in the animal body is possible, and this is absolutely the same in which its formation in plants takes place; it is a separation of oxygen from the elements of the food."

But this oxygen is not given out in the free state, because it meets in the organism with substances possessing the property of entering into combination with it. A remarkable connection is thus shown to exist between the formation of fat and the respiratory apparatus.

"The abnormal condition, which causes the deposit of fat in the animal body, depends, as was formerly stated, on the disproportion between the quantity of carbon in the food and that of oxygen absorbed by the skin and lungs. In the normal condition, the quantity of carbon given out is exactly equal to that which is taken in the food, and the body acquires no increase of weight from the accumulation of substances containing much carbon and no nitrogen.

"If we increase the supply of highly carbonized food, then the normal state can only be preserved on the condition that, by exercise and labor the waste of the body is increased, and the supply of oxygen augmented in the same proportion.

"The production of fat is always a consequence of a deficient supply of oxygen, for oxygen is absolutely indispensable for the dissipation of the excess of carbon in the food. This excess of carbon deposited in the form of fat, is never seen in the Bedouin or in the Arab of the desert, who exhibits with pride to the traveller, his lean, muscular, sinewy limbs, altogether free from fat; but in prisons and jails it appears as a puffiness in the inmates, fed, as they are, on a poor and scanty diet; it appears in the sedentary females of oriental countries; and finally, it is produced under the well-known condition of the fattening of domestic animals.

"The formation of fat depends on a deficiency of oxygen; but inthis process, in the formation of fat itself, there is opened up a new source of oxygen, a new cause of animal heat.

"The oxygen set free in the formation of fat is given out in combination with carbon or hydrogen; and whether this carbon and hydrogen proceed from the substance that yields the oxygen, or from other compounds, still there must have been generated by this formation of carbonic acid or water, as much heat as if an equal weight of carbon or hydrogen had been burned in air or in oxygen gas."

Professor Liebig shows by calculation, that every pound of carbon which obtains the oxygen nececsary to convert it into carbonic acid from substances which thereby pass into fat, must disengage as much heat as would raise the temperature of 200° lbs. of water by 70°—that is from 32° to 102°.

"Thus, in the formation of fat, the vital force possesses a means of counteracting a deficiency in the supply of oxygen, and consequently in that of the heat indispensable for the vital process."

"In some diseases, the starch, sugar, &c. of the food obviously do not undergo the changes which enable them to assist in respiration, and consequently to be converted into fat. Thus, in diabetes mellitus, the starch is only converted into grape sugar, which is expelled from the body without further change.

"In other diseases, as for example in inflammation of the liver, we find the blood loaded with fat and oil; and in the composition of the bile there is nothing at all inconsistent with the supposition that some of its constituents may be transformed into fat."

All aliments for man, according to our author, may be divided into two classes—*nitrogenized* and *non-nitrogenized*—the former, capable of conversion into blood, and called *the plastic elements of nutrition*, the latter incapable of this transformation, and which may be styled *elements of respiration.* Among the former he reckons vegetable fibrine, albumen, and caseine, and animal flesh and blood; and among the latter, fat, starch, gum, grape sugar, cane sugar, sugar of milk, pectine, bassorine, wine, beer, and spirits. The nitrogenized materials of food are identical in composition with the constituents of blood, and no nitrogenized substance the composition of which differs from that of fibrine, albumen and caseine is capable of supporting the vital process in animals. The blood, as has been remarked, must be supplied to them ready formed, and then their organism is prepared to form membranes, nerves, cartilages and the organic part of bones. But a question is suggested here as to the agency of gelatine in nutrition. It is not susceptible of conversion into blood, and it is known that dogs fed exclusively upon it starve, yet the gelatine of bones devoured by them entirely disappears. The same is true of the gelatine of the soup eaten by man, not a particle of which can be detected in any of his excretions. What becomes of it? The author thinks the opinion worthy of investigation, that it is again converted in the body into cellular tissue, membrane and cartilage, and may serve for the reproduction of such parts of these tissues as have been wasted, and for their growth. In the sick man, he remarks, the intensity of the vital force must be diminished, as well in the stomach, as in all other parts of the body. In this condition, it is well known that gelatinous matters, in a dissolved state exercise a beneficial influence on the state of the health. Given in a form adapted for assimilation, they serve to husband the vital force, just as may be done, in the case of the stomach, by due preparation of the food in general. And as the animal organism finds it difficult, if not impossible, to convert gelatine into blood, so the tissues chiefly composed of it, as the skin, cartilages, and membranes, yield slowly to the causes which effect rapid transformations in other parts of the system. While the fat in the body of a sick or starving man quickly disappears, and his muscular tissue is converted once more into blood, the tendons and membranes are but slightly diminished in size or altered in condition.

In part II, Professor Liebig treats of what he terms the *metamorphosis of the tissues*, under which head we find his theory of *digestion.* Albumen, fibrine and caseine, we have seen, are the elements out of which blood, and the other parts of the animal organism are formed, and these products of vegetable life, as has been shown, are identical in composition.

When exposed to a high temperature in a solution of caustic potash, they are decomposed, and by the action of acetic acid a precipitate is thrown down of the same precise character from each of these substances, which, on careful analysis, is found to contain the same organic elements, in exactly the same proportion, as the animal matters from which it is prepared. This product Mulder, to whom we owe the discovery of it, named *proteine*—the commencement and starting point of the animal tissues. The blood, or the constituents of the blood, appear to be compounds of this proteine with variable proportions of inorganic substances. Vegetables produce compounds of proteine, and out of these compounds the various parts of the animal body are developed by the vital force, with the aid of the oxygen of the atmosphere and of the elements of water.

"This proposition must be received as an undeniable truth, when we reflect on the development of the young animal in the egg of a fowl. The egg can be shown to contain no other nitrogenised compound except albumen. The albumen of the yolk is identical with that of the white; the yolk contains, besides, only a yellow fat, in which cholesterine and iron may be detected. Yet we see, in the process of incubation, during which no food and no foreign matter, except the oxygen of the air, is introduced, or can take part in the development of the animal, that out of the albumen, feathers, claws, globules of the blood, fibrine, membrane and cellular tissue, arteries and veins, are produced. The fat of the yolk may have contributed, to a certain extent, to the formation of the nerves and brain; but the carbon of this fat cannot have been employed to produce the organized tissues in which vitality resides, because the albumen of the white and of the yolk already contains, for the quantity of nitrogen present, exactly the proportion of carbon required for the formation of these tissues.

"The true starting-point for all the tissues is, consequently, albumen; all nitrogenized articles of food, whether derived from the animal or from the vegetable kingdom, are converted into albumen before they can take part in the process of nutrition."

Digestion, according to the author, is a chemical action similar to those processes of decomposition or transformation, known as putrefaction, fermentation, or decay, which latter he expresses by the word *eremacausis.* But it must be borne in mind that these chemical changes are not necessarily attended by the more striking phenomena of putrefaction and fermentation—there is not always present an offensive smell, nor is there of necessity any disengagment of gases. Starch is converted into sugar by the action of sulphuric acid without any escape of gas; fibrine and hard boiled white of an egg are transformed into liquids, by organic acids, or weak alkaline solutions, without the slightest change of properties except the solid form; and so there are numberless cases in which a complete chemical metamorphosis occurs without the smallest disengagement of gaseous matter. Expressed in the simplest form Professor Liebig remarks:

"Fermentation, or putrefaction, may be described as a process of transformation—that is, a new arrangement of the elementary particles, of a compound, yielding two or more new groups or compounds, and caused by contact with other substances, the elementary particles of which are themselves in a state of transformation or decomposition. It is a communication, or an imparting of a state of motion, which the atoms of a body in a state of motion are capable of producing in other bodies, whose elementary particles are held together only by a feeble attraction.

"Thus the clear gastric juice contains a substance in a state of transformation, by the contact of which with those constituents of the food which, by themselves, are insoluble in water, the latter acquire, in virtue of a new grouping of their atoms, the property of dissolving in that fluid. During digestion, the gastric juice, when separated, is found to contain a free mineral acid, the presence of which checks all further change. That the food is rendered soluble quite independently of the vitality of the digestive organs has been proved by a number of the most beautiful experiments. Food, enclosed in perforated metallic tubes, so that it could not come into contact with the stomach, was found to disappear as rapidly, and to be as perfectly digested, as if the covering had been absent; and fresh gastric juice, out of the body, when boiled white of egg, or muscular fibre, were kept in contact with it for a time at the temperature of the body, caused these substances to lose the solid form and to dissolve in the liquid."

"In the action of the gastric juice on the food, no other element takes a share, except the oxygen of the atmosphere and the elements of water. This oxygen is introduced directly into the stomach. During the mastication of the food, there is secreted into the mouth from organs specially destined to this function, a fluid, the saliva, which possesses the remarkable property of enclosing air in the shape of froth, in a far higher degree than even soapsuds. This air, by means of the saliva, reaches the stomach with the food, and there its oxygen enters into combination, while its nitrogen is given out through the skin and lungs. The longer digestion continues, that is, the greater resistance offered to the solvent action by the food, the more saliva, and consequently the more air enters the stomach. Rumination, in certain graminivorous animals, has plainly for one object a renewed and repeated introduction of oxygen; for a more minute mechanical division of the food only shortens the time required for solution."

The gastric juice is a product of the transformation of the stomach itself, and as yeast, which is nothing but vegetable fibrine, albumen or caseine in a state of decomposition, converts sugar into alcohol and carbonic acid; or as muscular fibre, when separated from the body, decomposes the peroxide of hydrogen, by virtue of the state of decomposition in which it is, so the gastric juice, the result of transformation imparts to the food a tendency to change—the insoluble matters become soluble, or are digested in consequence of an impulse which the gastric juice imparts. This is Professor Liebig's theory of digestion, and it is certainly at the same time a most simple and ingenious one.

And equally simple, according to this view, are the other transformations which take place in the system. Thus proteine passes into chondrine (the substance of the cartilages of the ribs,) by combining with the elements of water and oxygen; while in the formation of the serous membranes, nitrogen also has entered into combination. In horn, hair, &c., to the proteine, nitrogen and hydrogen are added in proportions to form ammonia. All the tissues of the body contain, for the same amount of carbon, more oxygen than the constituents of the blood, and hence during their formation oxygen, either from the atmosphere, or from the elements of water, must be added to the elements of proteine. These conclusions are followed by others still more curious.

"If it be true, that all parts of the body are formed and developed from the blood or the constituents of the blood, that the existing organs at every moment of life are transformed into new compounds under the influence of the oxygen introduced in the blood, then the animal secretions must of necessity contain the products of the metamorphosis of the tissues.

"If it be further true, that the urine contains those products of metamorphosis which contain the most nitrogen, and the bile those which are richest in carbon, from all the tissues which in the vital process have been transformed into unorganized compounds, it is clear that the elements of the bile and of the urine, added together, must be equal, in the relative proportion of these elements to the composition of the blood.

"The organs are formed from the blood, and contain the elements of the blood; they become transformed into new compounds, with the addition only of oxygen and water. Hence the relative proportion of carbon and nitrogen must be the same as in blood.

"If then we subtract from the composition of blood the elements of the urine, then the remainder, deducting the oxygen and water which have been added, must give the composition of the bile.

"Or if from the elements of the blood, we subtract the elements of the bile, the remainder must give the composition of urate of ammonia, or of urea and carbonic acid."

The transformed muscular fibre is to be looked for in the bile and urine, and is found, according to our author, in the urea of the latter and the choleic acid of the former. Urea degenerates into uric acid when the supply of oxygen is deficient, and it is a very remarkable fact that urinary calculi in the human subject vary with the amount of exercise taken by the individual, and consequently with the quantity of oxygen consumed. The calculi of oxalate of lime, or urate of ammonia are formed in persons in whom, from want of exercise, the supply of oxygen has been diminished. The calculi of patients who have resided in cities are succeeded by deposits containing more oxygen, when they go to the country; and finally when the person regains entire health the highest degree of oxidation takes place, and only carbonic acid and urea are formed in the kidneys.

The bile is a highly important product of the animal system, and the view which Professor Liebig takes of it, if it should be established by future researches, will enhance its interest. That element of the bile which contains the compounds resulting from the transformation of proteine,is choleic acid. In the carnivora it comes from the metamorphosed tissues; but not so in herbivorous animals. In them, other substances besides compounds of proteine must take part in the formation of bile, because the metamorphosis of tissues is not sufficient to account for the large quantity of this secretion formed. These substances, our author supposes, are sugar, starch, &c., which, uniting with the nitrogenized elements of food, supply the blood with the materials for forming it in such abundance—amounting, in the ox, to 37 lbs. a day. If starch be the chief ingredient, it happens by separating a quantity of oxygen, as when starch passes into fat. Starch can become bile in no other way; but the separation being admitted, its conversion into a compound intermediate between starch and fat offers no difficulty. Thus, Professor Liebig sums up:

"The transformation of the compounds of proteine present in the body is effected by means of the oxygen conveyed by the arterial blood, and if the elements of starch, rendered soluble in the stomach, and thus carried to every part, enter into the newly formed compounds, we have the chief constituents of the animal secretions and execretions; carbonic acid, the excretion of the lungs, urea and carbonate of ammonia, excreted by the kidneys, and choleic acid, secreted by the liver."

The remarks of the author on tea and coffee will be found to possess all the novelty which characterizes the views just presented. He says:

"We shall never, certainly, be able to discover how men were led to the use of the hot infusion of the leaves of a certain shrub (tea) or of a decoction of certain roasted seeds

(coffee.) Some cause there must be, which would explain how the practice has become a necessary of life to whole nations. But it is surely still more remarkable, that the beneficial effects of both plants on the health must be ascribed to one and the same substance, the presence of which in two vegetables, belonging to different natural families, and the produce of different quarters of the globe, could hardly have presented itself to the boldest imagination. Yet recent researches have shown in such a manner as to exclude all doubt, that caffeine, the peculiar principle of coffee, and theine, that of tea, are, in all respects, identical.

"It is not less worthy of notice, that the American Indian, living entirely on flesh, discovered for himself, in tobacco smoke, a means of retarding the change of matter in the tissues of his body, and thereby of making hunger more endurable; and that he cannot withstand the action of brandy, which acting as an element of respiration, puts a stop to the change of matter by performing the function which properly belongs to the products of the metamorphosed tissues. Tea and coffee were originally met with among nations whose diet is chiefly vegetable.

"Without entering minutely into the medicinal action of caffeine (theine,) it will surely appear a most striking fact, even if we were to deny its influence on the process of secretion, that this substance, with the addition of oxygen and the elements of water, can yield taurine, the nitrogenized compound peculiar to bile."

According to this view, *caffeine* may be considered *food for the liver*, supplying that organ with the elements necessary for the performance of its function. The author assuredly threads such mazes with a firm and confident step. No difficulties intimidate him. Now that we know the active principle upon which the virtues of opium, bark, &c., depend, "it would argue," he says, "only want of sense to consider the action of these substances inexplicable," and he marches up to the solution of their modus operandi in the following style:

"With respect to the action of the other nitrogenized vegetable principles, such as quinine, or the alkaloids of opium, &c., which manifests itself not in the processes of secretion, but in the phenomena of another kind, physiologists and pathologists entertain no doubt that it is exerted chiefly on the brain and nerves. This action is commonly said to be dynamic—that is, it accelerates, or retards, or alters in some way the phenomena of motion in animal life. If we reflect that this action is exerted by substances which are material, tangible and ponderable; that they disappear in the organism; that a double dose acts more powerfully than a single one; that, after a time, a fresh dose must be given, if we wish to produce the action a second time; all these considerations, viewed chemically, permit only one form of explanation; the supposition, namely that these compounds, by means of their elements, take a share in the formation of new, or the transformation of existing brain and nervous matter.

"Indeed, it cannot be considered merely accidental, that the composition of the most active remedies, namely, the vegetable alkaloids, cannot be shown to be related to that of any constituent of the body, except only the substance of the nerves and brain. All of these contain a certain quantity of nitrogen, and, in regard to their composition, they are intermediate between the compounds of proteine and the fats."

The action of certain other medicinal agents on the system is presented by the author in a novel point of view. It is known that many substances in very minute doses produce the most serious effects, and some of these, according to his views, effectuate the change by being themselves in a state of change. They originate an action which extends through the body, at the same time that their elements take no direct share in the changes which ensue. Their action on the blood is analogous to that of yeast on saccharine liquids, which is simply that of originating motion—of starting the fermentation. In this class, we find several poisons, of which we have perhaps an example in that which renders wounds in the dissecting room so dangerous. The sausage poison of Wurtemburg is considered a striking example. This poison is said to prove invariably fatal, and yet no poisonous substance can be detected in the sausages. They are in a state of fermentation, according to this theory, which the stomach fails to arrest, and which being communicated to the blood, spreads until every part capable of solution has been destroyed. By boiling, the poisonous sausage may be rendered wholesome. It is an interesting suggestion that miasms and contagions act upon the same principle—that they are organic matter, in a state of transformation which, introduced into the living body, act as a ferment, originating changes which extend throughout the system. But we have not space to enlarge upon it.

The third part of this work treats of the recondite laws of the phenomena of motion in the animal organism. It is principally of a speculative character, but some of the speculations are so interesting that we should like to lay them before our readers; the most that we can do, however, is to present a few of the author's deductions, which we do in his own words:

"For every portion of oxygen which enters into combination in the body, a corresponding portion of heat must be generated.

"The sum of force available for mechanical purposes must be equal to the sum of the vital forces of all tissues adapted to the change of matter.

"If, in equal times, unequal quantities of oxygen are consumed, the result is obvious, in an unequal amount of heat liberated, and of mechanical force.

"When unequal amounts of mechanical force are expended, this determines the absorption of corresponding and unequal quantities of oxygen.

"For the conversion of living tissues into lifeless compounds, and for the combination of oxygen with such constituents of the body as have an affinity for it, *time is required.*

"In a given time, only a limited amount of mechanical force can be manifested, and only a limited amount of heat can be liberated.

"That which is expended, in mechanical effects, in the shape of velocity, is lost in time; that is to say, the more rapid motions are, the sooner or the more quickly is the force exhausted.

"The sum of the mechanical force produced in a given time is equal to the sum of force necessary, during the same time, to produce the voluntary and involuntary motions; that is, all the force which the heart, intestines, &c., require for their motions is lost to the voluntary motions.

"The amount of azotized food necessary to restore the equilibrium between waste and supply, is directly proportional to the amount of tissues metamorphosed.

"The amount of living matter, which in the body loses the condition of life, is, in equal temperatures, directly proportional to the mechanical effects produced in a given time.

"The amount of tissue metamorphosed in a given time, may be measured by the quantity of nitrogen in the urine.

"The sum of the mechanical effects produced in two individuals, in the same temperature, is proportional to the amount of nitrogen in their urine; whether the mechanical force has been employed in voluntary or involuntary motions, whether it has been consumed by the limbs, or by the heart and other viscera.

"That condition of the body which is called *health*, includes the conception of an equilibrium among all the causes of waste and of supply; and thus animal life is recognized as the mutual action of both; and appears as an alternating destruction and restoration of the state of equilibrium.

"In regard to its absolute amount, the waste and supply of matter is, in the different periods of life, unequal; but, in the state of health, the available vital force must always be considered as a constant quantity, corresponding to the sum of living particles.

"Growth, or the increase of mass, stands, at every age, in a fixed relation to the amount of vital force consumed as moving power.

"The vital force, which is expended for mechanical purposes, is subtracted from the sum of the force available for the purpose of increase of mass.

"The active force, which is consumed in the body in overcoming resistance (in causing increase of mass,) cannot, at the same time, be employed to produce mechanical effects.

"Hence it follows necessarily, that when, as in childhood, the supply exceeds the waste of matter, the mechanical effects produced must be less in the same proportion.

"With the increase of mechanical effects produced, the capacity of increase of mass, or of the supply of waste in living tissues, must diminish in the same proportion.

"A perfect balance between the consumption of vital force for supply of matter and that for mechanical effects, occurs, therefore, only in the adult state. It is at once recognized in the complete supply of the matter consumed. In old age more is wasted; in childhood more is supplied than wasted.

"The force available for mechanical purposes in an adult man is reckoned, in mechanics, equal to 1-5th of his own weight, which he can move during eight hours, with a velocity of five feet in two seconds.

"If the weight of a man be 150 lbs., his force is equal to a weight of 30 lbs. carried by him to a distance of 72,000 feet. For every second his momentum of force is = 30+2.5 = 75

lbs.; and for the whole day's work, his momentum of motion is = 30 × 72,000 = 2,160,000.

"By the restoration of the original weight of his body, the man collects again a sum of force which allows him, next day, to produce, without exhaustion, the same amount of mechanical effects.

"*This supply of force is furnished in a seven hours' sleep.*"

Dr. Liebig's *theory of disease,* to which the last section of his book, but one, is devoted, is but little more than a speculation—another of the innumerable forms under which the human intellect has viewed a subject still veiled in obscurity. Some notion may be formed of his manner of treating this subject by the following extracts, which possess much interest apart from any hypothesis:

"To the observer, the action of a cause of disease exhibits itself in the disturbance of the proportion between waste and supply which is proper to each period of life. In medicine, every abnormal condition of supply or of waste, in all parts or in a single part of the body, is called disease.

"It is evident, that one and the same cause of disease will produce in the organism very different effects, according to the period of life; and that a certain amount of disturbance, which produces disease in the adult state, may be without influence in childhood or in old age. A cause of disease may, when it is added to the cause of waste in old age, produce death (annihilate all resistance on the part of the vital force;) while in the adult state it may produce only a disproportion between supply and waste; and in infancy, only an equilibrium between supply and waste (the abstract state of health).

"A cause of disease which strengthens the causes of supply, either directly or indirectly by weakening the action of the causes of waste, destroys, in the child and in the adult, the relative normal state of health; while in old age it merely brings the waste and supply into equilibrium.

"A child, lightly clothed, can bear cooling by a low external temperature without injury to health; the force available for mechanical purposes and temperature of its body increase with the change of matter which follows the cooling; while a high temperature, which impedes the change of matter, is followed by disease.

"On the other hand, we see, in hospitals and charitable institutions (in Brussels, for example) in which old people spend the last of life, when the temperature of the dormitory, in winter, sinks 2 or 3 degrees below the usual point, that by this slight degree of cooling the death of the oldest and weakest males, as well as females, is brought about. They are found lying tranquil in bed, without the slightest symptoms of disease, or of the usual recognizable causes of death.

"Practical medicine, in many cases, makes use of cold in a highly rational manner, as a means of exalting and accelerating, in an unwonted degree, the change of matter. This occurs especially in certain morbid conditions, in the substance of the centre of the apparatus of motion; when a glowing heat and a rapid current of blood toward the brain point out an abnormal metamorphosis of the brain. When this condition continues beyond a certain time, experience teaches that all motions in the body cease. If the change of matter be chiefly confined to the brain, then the change of matter, the generation of force, diminishes in all other parts. By surrounding the head with ice the temperature is lowered, but the cause of the liberation of heat continues; the metamorphosis which decides the issue of the disease, is limited to a short period. We must not forget, that the ice melts and absorbs heat from the diseased part; that if the ice be removed before the completion of the metamorphosis, the temperature again rises; that far more heat is removed by means of ice than if we were to surround the head with a bad conductor of heat. There has obviously been liberated in an equal time, a far larger amount of heat than in the state of health; and this is only rendered possible by an increased supply of oxygen, which must have determined a more rapid change of matter."

But, despairing of doing justice by extracts to these speculations, we hasten to conclude this article by giving an abstract of his theory of respiration.

The change in the color of the blood which takes place in the lungs is one of the most familiar facts in physiology. It seems to be connected, in some way, with the iron contained in the red globules, and which is found in no other constituent of the body. The external agent concerned in this change is oxygen. The globules of arterial blood retain their florid hue until they reach the capillaries where they lose it. Venous blood in contact with oxygen is reddened, and the change, in either case, depends upon the globules which have the power of combining with gases. These globules take no part in the process of nutrition, and it cannot be doubted, therefore, that their office is to aid in respiration. They contain iron, and no other metal can be compared with this for the remarkable properties of its compounds. Thus, the compounds of the *protoxide* of iron possess the power of depriving the other oxidized compounds of oxygen; while the compounds of *peroxide* of iron, under other circumstances, *give up* oxygen with the utmost facility. Now, in arterial blood the globules contain iron in the state of a peroxide—they are saturated with oxygen in the lungs. In the passage of the blood through the capillaries the peroxide of iron is reduced, by giving up a part of its oxygen to the carbon it there meets with, and in this way becomes a protoxide of iron, which combines with the carbonic acid just formed, and as a carbonate of iron passes on through the veins to the heart. When the globules reach the lungs, they will again take the oxygen they have lost; for every volume of oxygen absorbed they will liberate a corresponding volume of carbonic acid, and will be in a state to give off oxygen in the capillaries again. One purpose of the arterial globules is, to yield oxygen to certain constituents of the body, to produce the change of matter, determine the separation of living parts and their conversion into lifeless compounds, and aid in the formation of secretions and excretions. But the greater portion of the oxygen is employed in converting into oxidized compounds the newly-formed substances, which no longer make part of the living tissues. All the constituents present in venous blood, which have an attraction for oxygen, are converted, in the lungs, like the globules, into more highly oxidized compounds. From all of which it appears, that, in the animal organism, two processes of oxidation are going on—one in the lungs, the other in the capillaries. By means of the former, in spite of the degree of cooling, and of the increased evaporation which takes place there, the constant temperature of the lungs is kept up; while the heat of the rest of the body is supplied by the latter.

The frightful effects of sulphuretted hydrogen, and of prussic acid, when inspired, the author thinks are to be explained by the well-known action of these compounds on the preparations of iron. He says,

"Let us suppose that the globules lose their property of absorbing oxygen, and of afterwards giving up this oxygen and carrying off the resulting carbonic acid; such a hypothetical state of disease must instantly become perceptible in the temperature and other vital phenomena of the body. The change of matter will be arrested, while yet the vital motions will not be instantly stopped.

"The conductors of force, the nerves, will convey, as before, to the heart and intestines, the power necessary for their functions. This power they will receive from the muscular system, while, as no change of matter takes place in the latter, the supply must soon fail. As no change of matter occurs, no lifeless componds are separated, neither bile nor urine can be formed; and the temperature of the body must sink.

"This state of matters soon puts a stop to the process of nutrition, and, sooner or later, death must follow, but unaccompanied by febrile symptoms, which, in this case, is a very important fact.

"This example has been selected in order to show the importance and probable advantage of an examination of the blood in analogous diseased conditions. It cannot be, in the slighest degree, doubtful, that the function ascribed to the blood-globules may be considered as fully explained and cleared up, if, in such morbid conditions, we shall discover a change in their form, structure, or chemical characters, a change which must be recognizable by the use of appropriate reagents."

This view of respiration combines every quality that can be required in an hypothesis—it rests upon well-known observations, and explains all the phenomena of the process; it is probably, therefore, the true theory.

The appendix, the most elaborate part of Dr. Liebig's work, consisting of a great number of the most recent and exact analyses, and comprising the data upon which his conclusions rest, cannot, of course, be given, even in outline, in an analytical review. The work, we are sure, is destined to a wide and lasting popularity, and each reader will examine for himself the mass of curious and interesting details, upon which this beautiful system has been erected. To the chemist, the physiologist, the medical man, and the agriculturist, it is a volume of singular interest, shedding the brightest light upon every function of the animal system accessible to chemistry, and opening a path which has led to the most unexpected and astonishing results. Y.

